# rs4889 and rs5782018 polymorphisms of KISS1 gene as genetic predisposing factor for PCOS in Indonesian women

**DOI:** 10.12688/f1000research.168971.2

**Published:** 2026-02-02

**Authors:** Gita Pratama, Ririn R Febri, Mila Maidarti, Budi Wiweko, Asmarinah, Indah S Widyahening, Trinovita Andraini, Hartanto Bayuaji, Andon Hestiantoro, Mulyoto Pangestu

**Affiliations:** 1 Reproductive Immunoendocrinology Division, Department of Obstetrics and Gynecology, University of Indonesia Faculty of Medicine, Jakarta, Special Capital Region of Jakarta, Indonesia; 2 Yasmin IVF Clinic, Hospital Dr Cipto Mangunkusumo, Central Jakarta, Jakarta, Indonesia; 3 Human Reproduction, Infertility, and Family Planning Cluster, Indonesia Reproductive Medicine Research and Training Center, University of Indonesia Faculty of Medicine, Jakarta, Special Capital Region of Jakarta, Indonesia; 4 Department of Medical Biology, University of Indonesia Faculty of Medicine, Jakarta, Special Capital Region of Jakarta, Indonesia; 5 Department of Community Medicine, University of Indonesia Faculty of Medicine, Jakarta, Special Capital Region of Jakarta, Indonesia; 6 Department of Physiology, University of Indonesia Faculty of Medicine, Jakarta, Special Capital Region of Jakarta, Indonesia; 7 Department of Obstetrics and Gynecology, University of Padjadjaran Faculty of Medicine, Bandung, West Java, Indonesia; 8 Education Program in Reproduction and Development, Department of Obstetrics and Gynecology, School of Clinical Sciences, Monash University, Clayton, Victoria, Australia

**Keywords:** Haplotype, PCOS, polymorphism, rs4889, rs5780218

## Abstract

**Background:**

Dysregulation of the HPG axis in PCOS causes increased frequency and amplitude of gonadotropin-releasing hormone (GnRH) pulsatility in the hypothalamus. Single nucleotide polymorphisms (SNPs) in the KISS1 gene may be associated with altered neuroendocrine signaling in PCOS. The present study aims to evaluate the association between two *KISS1* polymorphisms (rs4889 and rs5780218), their haplotypes, and the odds of PCOS in Indonesian women.

**Methods:**

A cross-sectional study was conducted at Yasmin Clinic, dr. Cipto Mangunkusumo General Hospital, Indonesia, involving 60 women with PCOS and 60 healthy controls. Hormonal levels were assessed using ELISA, and genomic DNA was analyzed by Sanger sequencing. Demographic data were compared using independent t-tests, and chi-square tests were used for genotype and allele frequency analysis.

**Results:**

The genotypic distribution of rs4889 was significantly different between the PCOS and control groups (p<0.05), where the distribution of mutant genotype GG was higher in PCOS than in control (18.3% and 1.7%, respectively). The allele distribution of rs4889 and rs5782018 KISS1 SNPs were significantly different between both groups (p<0.01 and p<0.05, respectively). The rs4889 polymorphism was significantly different between the PCOS and control groups for the codominant and recessive models (p<0.01). Moreover, the rs5780218 polymorphism was significantly different between the PCOS and control groups for the codominant and dominant models (p<0.05). From the haplotype analysis, the G-CT haplotype was significantly different, with an OR value of 2.57 (1.33–4.96, p=0.0057).

**Conclusions:**

*KISS1* rs4889 and rs5780218 polymorphisms, as well as the G–CT haplotype, are associated with increased odds of PCOS in Indonesian women. These findings support a potential role of upstream neuroendocrine genetic variation in PCOS susceptibility; however, causal inferences cannot be drawn from this cross-sectional study.

**List of abbreviations**
AIC: Akaike information criteria
ARC: Arcuate
ASRM: American Society for Reproductive Medicine
AVPV: Anteroventral periventricular
BIC: Bayesian information criteria
CI: Confidence interval
DNA: Deoxyribonucleic acid
ELISA: Enzyme-linked immunoassay
ESHRE: The European Society of Human Reproduction and Embryology
FAI: Free androgen index
FSH: Follicle-stimulating hormone
GnRH: Gonadotropin-releasing hormone
GWAS: Genome-wide association study
HOMA-IR
: Homeostatic model assessment for insulin resistance
HPG: Hypothalamic-pituitary-gonadal
HWE: Hardy-Weinberg equilibrium
LD: Linkage disequilibrium
LH: Luteinizing hormone
mRNA: Messenger ribonucleic acid
OR: Odds ratio
PCOS: Polycystic ovary syndrome
PCR: Polymerase chain reaction
Rs: Restriction site
SHBG: Sex-hormone binding globulin
SNP: Single nucleotide polymorphism
T: Testosterone

## Introduction

Polycystic ovary syndrome (PCOS) is a common and complex endocrine disorder characterized by clinical or biochemical hyperandrogenism, oligo-anovulation, and polycystic ovaries; it affects 6% to 10% of women of reproductive age. ^1^ The etiology of PCOS is not entirely understood. The complex pathogenesis involves hypothalamic-pituitary-gonadal (HPG) axis disturbances in gonadotropin secretion and increased LH levels. ^2, 3^ Several previous studies have focused on investigating the genetic factors of the HPG axis related to PCOS and reported a disruption in the function of the HPG axis, causing increased frequency and amplitude of gonadotropin-releasing hormone (GnRH) pulsatility in the hypothalamus. ^4^ The KISS1 gene is considered to have an essential role in regulating gonadotropin secretion in the HPG axis and may be involved in the etiology of PCOS.

KISS1 is located at 1q32 and encodes a premature 145-amino-acid protein. The major product of the KISS1 gene, kisspeptin, is a 54-amino acid peptide that was first identified as a melanoma metastasis-suppressor gene. ^5^ The binding of kisspeptin with its receptor (GPR54) in GnRH neurons of the hypothalamus activates the HPG axis, causing stimulation of gonadotropin release into the portal circulation. Gonadotropin binds the GnRH receptors in the anterior pituitary gland, consequently releasing and stimulating the activity of luteinizing hormone (LH) and follicle-stimulating hormone (FSH). ^5, 6^ Ovulatory dysfunction of PCOS is reflected biochemically through excessive production of LH and a low or normal level of FSH from the anterior pituitary gland. ^7^ A study showed potential pathophysiology involving KNDy neurons, which increased the NKB levels, decreased dynorphin levels, and elevated kisspeptin secretion, which resulted in increased LH levels and decreased FSH levels. ^8^ Kisspeptin may interfere with LH production in PCOS and disrupt ovarian function, prompting androgen hypersecretion in the theca interna cells of the ovarian follicles. ^7^ Although the prior studies have revealed the correlation between kisspeptin and the HPG axis, whether the serum kisspeptin concentration is higher in PCOS women compared to the general population remains inconclusive.

Single nucleotide polymorphisms (SNPs) in the KISS1 gene could lead to the disruption of GnRH secretion by dysregulating HPG axis function and increasing the development of various diseases. Several studies have indicated that some SNPs in KISS1 may play a vital role in the etiopathogenesis of PCOS. ^4, 5, 9^ The current study determined the impact of KISS1 gene polymorphisms and haplotypes on the development of PCOS.

## Methods

### Subjects

**Figure 1.**
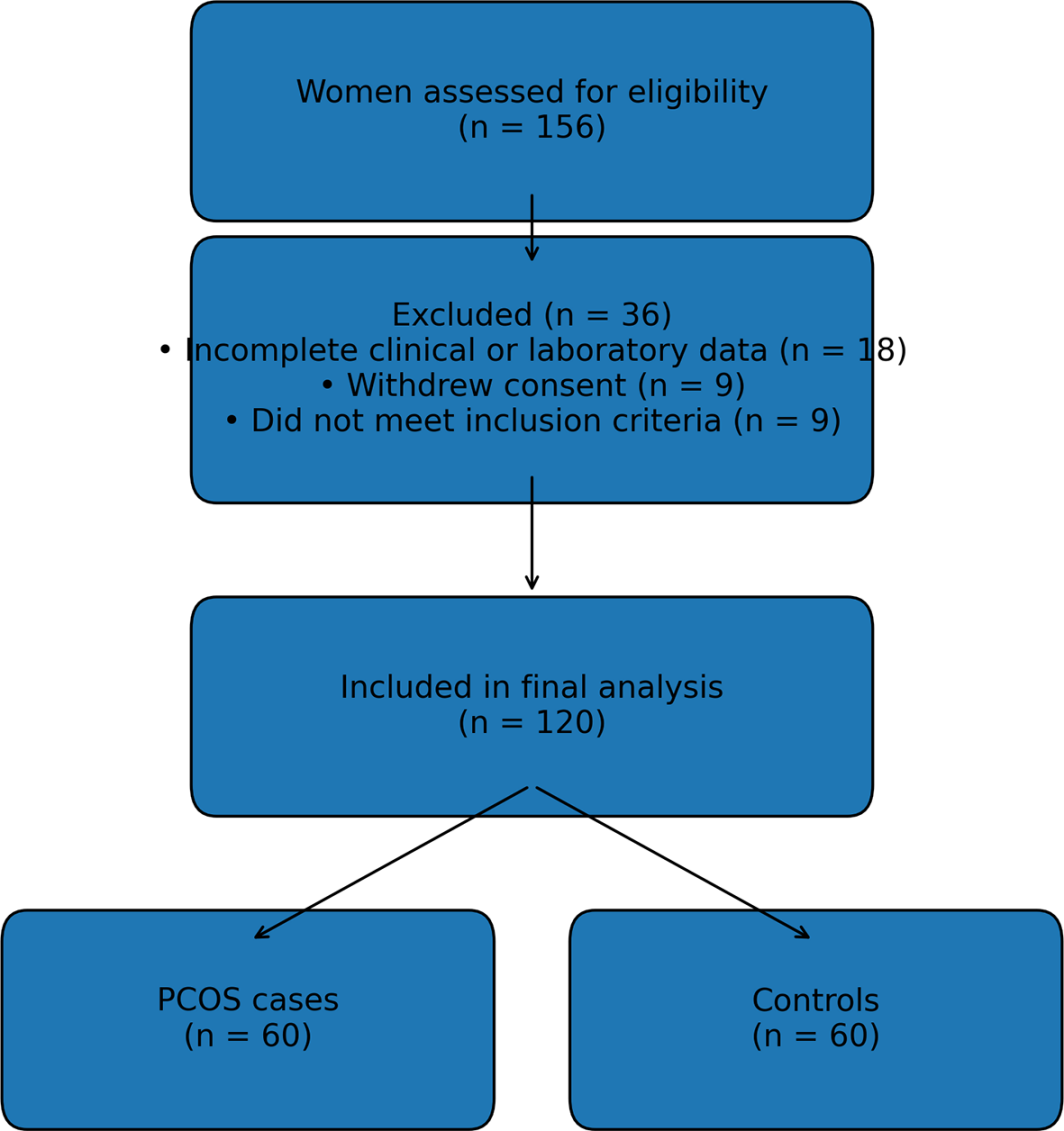
Flow diagram of the participants.

The study recruited 80 women with PCOS and 76 control women who attended a clinic in Jakarta, Indonesia. However, only sixty subjects for each group met the inclusion criteria. Written informed consent was obtained from each subject. All subjects were self-reported as having Indonesian ethnic origin. Sixty women with PCOS cases were diagnosed according to the Revised 2003 consensus on diagnostic criteria (Rotterdam ESHRE/ASRM-Sponsored PCOS Consensus Workshop Group 2004), i.e., any two of the following three criteria: oligo- or anovulation, clinical and/or biochemical signs of hyperandrogenism, and polycystic ovary morphology determined by ultrasonography. ^10^ Oligo- or anovulation was determined by self-reported irregular menstrual cycles (menstruation occurring with a frequency more than 35 days in the year before enrollment). Hyperandrogenism was defined with hirsutism using a Ferriman-Gallwey score of more than eight, and/or the serum level of total testosterone greater than 60 ng/dl. Patients with any other cause of oligomenorrhea or hyperandrogenism, such as congenital adrenal hyperplasia, Cushing’s syndrome, hypothyroidism, or significant elevations in serum prolactin, were excluded. The transvaginal ultrasonography was used to assess the ovarian morphology for further stratified evidence of women with PCOS. A participant flow diagram is provided below in Figure 1.

Sixty healthy women with regular menstrual cycles and without hyperandrogenism were recruited as controls. In addition to meeting these recruiting criteria, no subjects had taken medications known to alter the normal function of the HPG axis in the last 3 months. For all subjects, on the 3 ^rd^ to 5 ^th^ day of the menstrual cycle after overnight fasting, 5 ml whole-blood samples were obtained for both SNP analysis and reproductive hormone measurement. In subjects with PCOS, fasting blood insulin and fasting blood glucose were measured to determine insulin resistance.

### Analysis of the rs4889 and rs5780218 KISS1 genes

Genomic DNA was isolated from the whole blood of women with PCOS and the control individuals using the Geneaid™ DNA Isolation Kit (Geneaid Biotech Ltd., New Taipei City, Taiwan) according to the manufacturer’s protocol. The rs4889 and rs5780218 variants of the KISS1 gene were amplified with polymerase chain reaction (PCR). The sequences of the rs4889 primers were 5’-CTA AGG TGA TCG TGG TT-3’ (forward primer) and 5’-CAG TTG TAG TTC GGC AGG T-3’ (reverse primer) with the product size 388 bp. The sequences of the rs5780218 primers were 5’-AAG GTG CCA TGC TCT TCA G-3’ (forward primer) and 5’-GGA TGC ATC TGT CCG TCT TAG-3’ (reverse primer) with the product size 332 bp. PCR amplification was carried out in a final volume of 20 μl containing 10 μl 2x SensiFAST SYBR ^®^ No-ROX mix (Meridian Bioscience, Tennessee, USA), 10 μM of each primer, and 50 ng of genomic DNA. PCR was performed in a SEDI Thermal Cycler (Wealtec Bioscience Co., Ltd., New Taipei City, Taiwan) with the following conditions: 95°C for 2 min, followed by 35 cycles of 15 sec at 95°C, 15 sec at 56°C, and 10 sec at 72°C. The single-band PCR products were visualized on a 1.6% agarose gel with GelRed ^®^ Nucleic Acid gel staining (Biotium Inc., Fremont, CA, USA). For those subjects with a single band, the PCR products were directly sequenced using an ABI PRISM 3100 Genetic Analyzer (ABI, Applied Biosystems, Foster City, CA, USA), and each set of reactions was run with positive and negative controls.

### Hormone measurements

Levels of total testosterone (T), follicle-stimulating hormone (FSH), luteinizing hormone (LH), insulin, prolactin and sex-hormone binding globulin were measured using TOSOH (Tosoh India Pvt. Ltd., Mumbai, India) according to the manufacturer’s protocols. The level of kisspeptin was measured using a Human KISS1 (Kisspeptin 1) ELISA kit (Elabscience, Texas, USA). Samples were processed under standardized preanalytical conditions and stored at −80°C until analysis.

### Statistical analysis

The data are presented as the mean ± SE (standard error) for demographic (age, body mass index (BMI), waist circumference, hip circumference, and waist-hip ratio) and endocrine (kisspeptin, prolactin, testosterone, SHBG, free androgen index (FAI), LH, FSH, LH-FSH ratio, insulin, fasting blood glucose, and homeostatic model assessment for insulin resistance (HOMA-IR)) characteristics. The independent t test was used to determine the differences in the variables between two groups. The chi-square test was carried out for the statistical analysis of the genotype and allele distribution of the polymorphisms, as well as to verify the Hardy-Weinberg Equilibrium (HWE). A value of p < 0.05 was considered to indicate significance. The statistical analysis was performed using Prism 5 software (GraphPad Software).

The sanger sequencing data was analyzed using the Tracy’s web application (available at https://www.gear-genomics.com). The logistic regression model was calculated using the SNPStats program (available at https://bioinfo.iconcologia.net/index.php?module=Snpstats). It was also used to evaluate the association between polymorphisms and the development of PCOS. The effect of polymorphisms was evaluated by the following models: (1) codominant (wild-type homozygote x heterozygote x polymorphic homozygote); (2) dominant (wild-type homozygote x heterozygote + polymorphic homozygote); (3) recessive (polymorphic homozygote x wild-type + heterozygote homozygote); and (4) overdominant (wild-type homozygote + polymorphic homozygote x heterozygote). The best inheritance model was assessed using the Akaike information criteria (AIC) and the Bayesian information criteria (BIC), and the model with the lowest values was considered the best fit. The haplotype frequencies for multiple loci and the standardized disequilibrium coefficient (D’) for pairwise linkage disequilibrium (LD) from KISS1 gene polymorphisms were inferred using the SNPStats program, checking the estimated population frequency of haplotypes. The LD level was defined as strong LD (D’ > 0.8), moderate LD (0.4 < D’ ≤ 0.8), and weak LD (D’ ≤ 0.4). ^11^ The results are presented as odds ratios (ORs) and 95% confidence intervals (CIs: 95%), with the level of statistical significance defined as p < 0.05.

## Results

A total of 120 women were included in the final analysis, comprising 60 women with PCOS and 60 control women. Median age did not differ significantly between the control group (24.0 years, range 17.0–40.0) and the PCOS group (26.0 years, range 18.0–35.0; p = 0.087). There were no statistically significant differences between groups in body mass index, waist circumference, hip circumference, or waist–hip ratio (all p > 0.05).

Circulating kisspeptin levels were not significantly different between groups (control: median 259.77 pg/mL [20.65–1580.36] vs PCOS: median 111.26 pg/mL [20.73–1442.05]; p = 0.128). Women with PCOS had significantly higher total testosterone levels, free androgen index (FAI), luteinizing hormone (LH), LH/FSH ratio, fasting insulin, and HOMA-IR, and significantly lower sex hormone–binding globulin (SHBG) levels compared with controls (all p < 0.05). Follicle-stimulating hormone (FSH), prolactin, and fasting blood glucose levels did not differ significantly between groups. These data are summarized in Table 1.

**Table 1.** Demographic and endocrine characteristics of study subjects.

Characteristics	Control (n = 60)	PCOS (n = 60)	p value
Age (years)	24.00 (17.00–40.00)	26.00 (18.00–35.00)	0.087
Body mass index (kg/m ^2^)	23.85 (18.50–39.40)	24.45 (18.50–42.30)	0.144
Waist circumference (cm)	76.00 (61.00–112.00)	79.50 (61.00–120.00)	0.075
Hip circumference (cm)	95.00 (77.00–135.00)	97.75 (78.00–145.00)	0.116
Waist-hip ratio	0.80 ± 0.01	0.81 ± 0.01	0.646
Kisspeptin (pg/ml)	259.77 (20.65–1580.36)	111.26 (20.73–1442.05)	0.128
Testosterone total (ng/dl)	40.03 (2.93–559.36)	71.01 (7.80–184.08)	0.000 *
SHBG (nmol/l)	66.59 (7.53–174.65)	37.00 (14.12–429.52)	0.000 *
Free androgen index (FAI)	2.41 (0.17–30.38)	5.96 (0.54–32.02)	0.000 *
Prolactin (ng/ml)	8.5 (4.10–40.80)	8.1 (2.50–79.40)	0.213
LH (mIU/ml)	4.55 (0.60–172.00)	10.20 (2.30–28.60)	0.000 *
FSH (mIU/ml)	5.75 (1.40–21.70)	7.1 (1.90–10.90)	0.051
LH-FSH ratio	0.79 (.15–7.93)	1.45 (0.52–3.25)	0.000 *
Insulin (mIU/l)	7.30 (2.60–37.80)	10.30 (2.30–135.30)	0.022 *
Fasting blood glucose (mg/dl)	92.00 (74.00–118.00)	94.00 (74.00–125.00)	0.270
HOMA-IR	1.70 (0.58–86.00)	2.48 (0.52–32.74)	0.032 *

T-independent test

All data are presented as the mean ± SE.

* p < 0.05.

**Figure 2.**
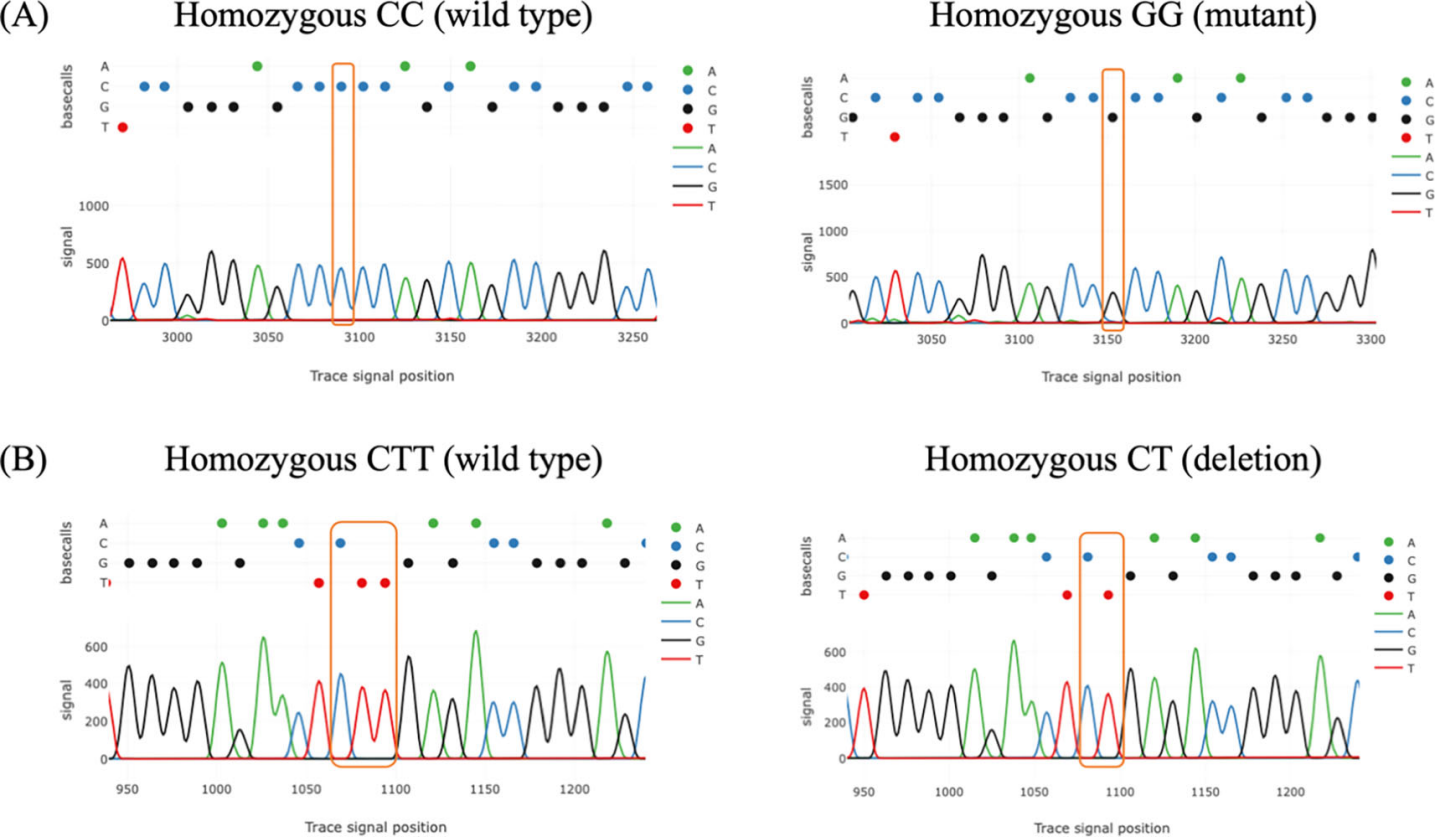
Chromatograms of PCR amplicon sequencing showing the DNA change in women with PCOS compared to control. (A) The comparison between wild type and mutant genotypes of rs4889. (B) The comparison between wild type and deletion genotypes of rs5780218.

All samples were successfully genotyped for rs4889 and rs5780218. Genotype distributions in the control group were in Hardy–Weinberg equilibrium for both polymorphisms (p > 0.05). Tracy’s web application was used for analyzing chromatogram trace file from the sanger sequencing data. The result of chromatogram for rs4889 and rs5780218 was shown in Figure 2. For rs4889, the distribution of genotypes differed significantly between PCOS and control groups (p = 0.006). The GG genotype was more frequent in women with PCOS (18.3%) than in controls (1.7%). The frequencies of CC and CG genotypes were 31.7% and 50.0% in the PCOS group, and 46.7% and 51.6% in the control group, respectively ( Table 2).

**Table 2.** Genotype frequencies of rs4889 and rs5780218 of the KISS1 gene.

SNPs	Genotypes	Frequency			p value
		Control n (%)	PCOS n (%)	Total n (%)	
rs4889	CC	28 (46.7)	19 (31.7)	47 (39.2)	0.006 *
	CG	31 (51.6)	30 (50.0)	61 (50.8)	
	GG	1 (1.7)	11 (18.3)	12 (10.0)	
	Total	60 (100.0)	60 (100.0)	120 (100.0)	
rs5780218	CTT/CTT	23 (38.4)	12 (20.0)	35 (29.2)	0.065
	CTT/CT	31 (51.6)	37 (61.7)	68 (56.7)	
	CT/CT	6 (10.0)	11 (18.3)	17 (14.1)	
	Total	60 (100.0)	60 (100.0)	120 (100.0)	

Chi-square test.

* p < 0.05.

For rs5780218, genotype distributions did not differ significantly between groups (p = 0.065). The frequencies of the CTT/CTT, CTT/CT, and CT/CT genotypes in the PCOS group were 20.0%, 61.7%, and 18.3%, respectively, compared with 38.4%, 51.6%, and 10.0% in controls ( Table 2).

Allele frequency analysis showed significant differences between PCOS and control groups for both polymorphisms. For rs4889, the G allele frequency was higher in the PCOS group than in controls (45.0% vs 27.5%), corresponding to an unadjusted odds ratio (OR) of 2.16 (95% CI 1.26–3.70; p = 0.0005). For rs5780218, the CT allele frequency was higher in the PCOS group than in controls (50.0% vs 35.8%), with an OR of 1.79 (95% CI 1.07–3.00; p = 0.027) ( Table 3).

**Table 3.** Allele frequencies of rs4889 and rs5780218 of the KISS1 gene.

SNPs	Allele	Frequency		Total n (%)	Odds ratio (95% CI)	p value
		Control n (%)	PCOS n (%)			
rs4889	C	87 (72.5)	66 (55.0)	153 (63.7)	2.157 (1.259–3.696)	0.0005 *
	G	33 (27.5)	54 (45.0)	87 (36.3)		
	Total	120 (100.0)	120 (100.0)	240 (100.0)		
rs5780218	CTT	77 (64.2)	60 (50.0)	137 (57.1)	1.791 (1.068–3.003)	0.027 *
	CT	43 (35.8)	60 (50.0)	103 (42.9)		
	Total	120 (100.0)	120 (100.0)	240 (100.0)		

Chi-square test.

* p < 0.05.

Logistic regression analyses were performed under multiple inheritance models ( Table 4). For rs4889, a significant association with PCOS was observed under the codominant model (p = 0.0017) and the recessive model (p < 0.001). Under the recessive model, carriers of the GG genotype had higher odds of PCOS compared with CC/CG genotypes (OR 14.75, 95% CI 1.85–117.51). The recessive model showed the lowest AIC and BIC values, indicating the best fit among tested models.

**Table 4.** Associations of the rs4889 and rs5780218 polymorphisms of the KISS1 gene with PCOS.

Model	Genotype	Control n (%)	PCOS n (%)	OR (95% CI)	p value	AIC	BIC
rs4889							
Codominant	C/C	28 (46.7)	19 (31.7)	1.00	0.0017 *	159.6	167.9
	C/G	31 (51.7)	29 (48.3)	1.38 (0.64-2.98)			
	G/G	1 (21.7)	12 (20)	17.68 (2.12–147.56)			
Dominant	C/C	28 (46.7)	19 (31.7)	1.00	0.092	167.5	173.1
	C/G-G/G	32 (53.3)	41 (68.3)	1.89 (0.90–3.97)			
Recessive	C/C-C/G	59 (98.3)	48 (80)	1.00	0.000 * *	158.3	163.8
	G/G	1 (1.7)	12 (20)	14.75 (1.85–117.51)			
Overdominant	C/C-G/G	29 (48.3)	31 (51.7)	1.00	0.72	170.2	175.8
	C/G	31 (51.7)	29 (48.3)	0.88 (0.43–1.79)			
rs5780218							
Codominant	CTT/CTT	24 (40.0)	12 (20.0)	1.00	0.036 *	165.7	174.1
	CTT/CT	30 (50.0)	36 (60.0)	2.40 (1.03–5.59)			
	CT/CT	6 (10.0)	12 (20.0)	4.00 (1.20–13.28)			
Dominant	CTT/CTT	24 (40.0)	12 (20.0)	1.00	0.016 *	164.6	170.1
	CTT/CT-CT/CT	36 (60.0)	48 (80.0)	2.67 (1.18–6.03)			
Recessive	CTT/CTT-CTT/CT	54 (90.0)	48 (80.0)	1.00	0.12	168.0	173.5
	CT/CT	6 (10.0)	12 (20.0)	2.25 (0.78–6.46)			
Overdominant	CTT/CTT-CT/CT	30 (50.0)	24 (40.0)	1.00	0.27	169.1	174.7
	CTT/CT	30 (50.0)	36 (60.0)	1.50 (0.73–3.09)			

Chi-square test.

* p < 0.05.

For rs5780218, significant associations were observed under the codominant (p = 0.036) and dominant (p = 0.016) models. Under the dominant model, carriers of at least one CT allele (CTT/CT or CT/CT) had higher odds of PCOS compared with the CTT/CTT genotype (OR 2.67, 95% CI 1.18–6.03). The dominant model demonstrated the lowest AIC and BIC values.

Multivariable logistic regression analyses adjusting for age, body mass index, and HOMA-IR showed effect estimates consistent in direction with unadjusted analyses, although confidence intervals widened, reflecting limited power for rare genotypes.

Linkage disequilibrium analysis between rs4889 and rs5780218 demonstrated strong LD (D′ = 0.864). Haplotype analysis identified four haplotypes. The C–CTT haplotype was the most frequent and served as the reference. The G–CT haplotype was more frequent in women with PCOS than in controls (0.403 vs 0.298) and was associated with increased odds of PCOS (OR 2.57, 95% CI 1.33–4.96; p = 0.0057). Other haplotypes (C–CT and G–CTT) were not significantly associated with PCOS ( Table 5).

**Table 5.** Construction of the haplotypes of the two SNPs in the KISS1 gene.

No	rs4889	rs5780218	Control Freq.	Case Freq.	Total Freq.	OR (95% CI)	p value
1	C	CTT	0.606	0.429	0.542	1.00	-
2	G	CT	0.298	0.403	0.3254	2.57 (1.33–4.96)	0.0057 *
3	C	CT	0.069	0.138	0.0996	1.38 (0.56–3.37)	0.49
4	G	CTT	0.027	0.030	0.033	1.81 (0.36–9.18)	0.48

* The values have significant differences (p < 0.05).

## Discussion

Impaired negative feedback from ovarian steroid hormones to the GnRH neuronal network is a crucial pathological feature of PCOS. ^12^ This alteration likely drives abnormalities in the neuroendocrine axis, which regulates fertility and primarily mediates downstream ovarian dysfunction. Women with PCOS demonstrate persistently rapid GnRH pulse frequency. This leads to LH pulsatility elevation and FSH deficiency and contributes to increases in LH concentrations and LH:FSH ratios, which are typical of PCOS. ^13^ Since GnRH neurons do not have specific receptors for steroid hormones, it is essential to understand the pathways involved in dysfunctional GnRH release. Kisspeptin is a potential candidate for stimulating the activity of GnRH and thus increasing LH levels.

We found that the serum level of kisspeptin was lower in women with PCOS than in healthy control individuals. In contrast with our results, several studies showed a higher expression level of kisspeptin in PCOS. ^7, 14– 16^ The low level of kisspeptin is possibly caused by the different pathways determining the relationship between kisspeptin and reproduction control that involve the complex mechanisms that regulate HPG function and reproductive physiology. ^17^ In the hypothalamus, kisspeptin cells are located in the anteroventral periventricular (AVPV) and arcuate (ARC) nuclei. ^18^ In the ARC, the expression of KISS1 mRNA is inhibited by testosterone. Moreover, the free androgen index (FAI) is correlated with the kisspeptin level. In this study, the expression levels of testosterone and FAI were higher in PCOS patients than in control individuals. Then, we suspected that a low kisspeptin level was associated with increased testosterone levels and FAI values in this study.

Excess levels of androgen and FAI in PCOS are fundamental hormonal problems that might be caused by several upstream factors in neuroendocrine regulation, including the alteration of LH pulsatility. ^19^ Our results demonstrated higher levels of LH, a lower FSH level, and a higher LH to FSH ratio in PCOS women than in control individuals. An elevated LH pulse frequency, increased serum LH concentration, and high LH-to-FSH ratio are common clinical characteristics of PCOS. These phenomena consequently affect the downstream pathway in the ovary; this includes the over synthesis of androgen in theca cells, leading to excess androgen production. ^20^ Low levels of FSH contribute to follicular arrest, preventing ovulation in PCOS women. ^21^

Multiple factors are more likely to cause PCOS since several mechanisms are involved in developing the disease. ^3^ However, due to the heterogeneity and uncertain etiology of PCOS, genes involved in PCOS are challenging to identify. Several genes have been identified as susceptible loci in previous genome-wide association studies (GWAS), including genes associated with steroid biosynthesis and gonadotropic secretion. ^22^ KISS1 may also be subjected to mutations and polymorphisms. ^6^ The neuropeptide kisspeptin induces the activity of GnRH neurons, consequently releasing GnRH. Since KISS1 acts in the upstream pathway of GnRH, disruption in KISS1 might affect GnRH signaling, especially in PCOS.

The involvement of the alterations in the hypothalamic-pituitary-gonadal (HPG) axis, especially the polymorphisms of KISS1 gene loci, was evaluated. Using Sanger sequencing, we detected two sites of polymorphisms, rs4889 and rs5780218. The results revealed a significantly higher frequency of the homozygous mutant genotype GG of rs4889 in PCOS patients than in control individuals, but no significant differences in the genotype distribution of rs5780218 were observed. The rs4889 polymorphism changes a CCC codon to CGC, resulting in the substitution of the amino acid proline by arginine, consequently disrupting the ability of kisspeptin to find its receptor. A previous study on the Saudi population also demonstrated similar results to our finding in the genotype distribution of rs4889. ^9^ In contrast with our results, a study in the Sri Lankan population by Branavan et al. demonstrated no association of rs4889 and rs5780218 with PCOS. ^23^

The frequencies of the G allele in rs4889 and CT in rs5780218 were also significantly higher in women with PCOS than in healthy control individuals, suggesting a strong association between KISS1 polymorphisms and an increased risk of PCOS. In addition, our findings also revealed that rs4889 C > G was linked in the codominant and recessive models. Moreover, rs5782018 CTT > CT was connected with the codominant and dominant model. Genetic variation is best described by groups of associated polymorphisms, called haplotypes. ^24^ Haplotype analysis was performed to find the most susceptible haplotype associated with PCOS. Our present data found that the presence of G-CT haplotypes was associated with an increased risk of PCOS. In summary, our results suggest that the KISS1 rs4889 and rs5780218 gene variants could be genetic predisposing factors for PCOS. Furthermore, we found that the risk haplotype G-CT in the KISS1 gene correlated with PCOS.

## Conclusions

KISS1 rs4889 and rs5780218 polymorphisms and the G–CT haplotype are associated with increased odds of PCOS in Indonesian women. These findings support further investigation of upstream neuroendocrine genetic contributors to PCOS pathophysiology in larger and longitudinal studies.

## Ethics approval

This study was conducted in accordance with the principles of the Declaration of Helsinki ( *https://www.wma.net/policies-post/wma-declaration-of-helsinki/* *).* Ethical approval was obtained from the Ethics Committee of the Faculty of Medicine, University of Indonesia – Cipto Mangunkusumo Hospital (Approval No. 23-12-2107) prior to commencement of the study.

## Data Availability

Open Science Framework: DATA - rs4889 and rs5782018 polymorphisms of KISS1 gene as genetic predisposing factor for PCOS in Indonesian women. https://doi.org/10.17605/OSF.IO/KFM6C. ^25^ The dataset underlying this study are available in the Open Science Framework (OSF) repository and can be accessed at: https://osf.io/kfm6c/ (DOI: 10.17605/OSF.IO/KFM6C). ^25^ All data are shared under a Creative Commons Attribution 4.0 International Public License (CC-BY 4.0 license). The repository includes the raw and processed data used in the analyses, including values underlying summary statistics, data used to generate figures, and any points extracted from images for analysis. No restrictions apply to data access.
