# Supplementary material for: rs4889 and rs5782018 polymorphisms of KISS1 gene as genetic predisposing factor for PCOS in Indonesian women
Source: F1000Res. 2026 Feb 2;14:889. Originally published 2025 Sep 9. [Version 2] doi: 10.12688/f1000research.168971.2 (PMC13182897; doi:10.12688/f1000research.168971.2)
Supplement: Peer review history

# Reviewer report: Reviewer response for version 2

**DOI:** 10.5256/f1000research.194949.r468339

**Published:** 2026-04-15

**Authors:** Mohd Faizal Ahmad

**Affiliations:** 1 Department of Obstetrics & Gynecology, Faculty of Medicine, National University of Malaysia, Kuala Lumpur, Malaysia

Strengths

 This study explores an important and timely topic by examining KISS1 gene polymorphisms in relation to PCOS, focusing on how these genes may affect the neuroendocrine regulation of the HPG axis. The link between kisspeptin signaling, GnRH pulsatility, and PCOS is well explained and fits with current scientific knowledge. Including an Indonesian cohort adds valuable genetic data from a group that is often underrepresented in this research area. The study uses Sanger sequencing for genotyping and analyzes genotypes, allele frequencies, inheritance models, and haplotypes, which strengthens its methods. Finding a significant G–CT haplotype linked to higher odds of PCOS adds depth beyond just looking at single SNPs. The clinical and hormonal profiles of participants also match classic PCOS features, which supports the reliability of the data.

 Weaknesses

 The main weakness of this study is its small sample size, which reduces statistical power and may lead to overestimation of effect sizes, especially for rare genotypes such as rs4889 GG. The study lacks a power calculation, which makes it harder to assess the strength of the results. Testing multiple genetic models without correcting for multiple comparisons could lead to false positives. Details on genotyping quality control, such as call rates and replicate agreement, are not fully reported, which affects the reproducibility of the findings. The study also uses self-reported menstrual history to diagnose oligo-ovulation, which could introduce recall bias, and relies on immunoassay-based testosterone measurements without discussing their limitations. The conclusions about kisspeptin are somewhat overstated, given the lack of significant differences. There are also some inconsistencies in how statistics are reported and minor issues with genetic terminology. Finally, the analyses do not clearly adjust for potential confounders such as BMI and insulin resistance, and using self-reported ethnicity could introduce population stratification bias.

Is the work clearly and accurately presented and does it cite the current literature?

Yes

If applicable, is the statistical analysis and its interpretation appropriate?

Yes

Are all the source data underlying the results available to ensure full reproducibility?

Yes

Is the study design appropriate and is the work technically sound?

Yes

Are the conclusions drawn adequately supported by the results?

Yes

Are sufficient details of methods and analysis provided to allow replication by others?

Yes

Reviewer Expertise:

Reproductive & Oncofertility

I confirm that I have read this submission and believe that I have an appropriate level of expertise to confirm that it is of an acceptable scientific standard.

# Reviewer report: Reviewer response for version 1

**DOI:** 10.5256/f1000research.186221.r445139

**Published:** 2026-01-05

**Authors:** Mushtak T. S. Al-Ouqaili

**Affiliations:** 1 Department of Microbiology, University of Anbar, College of Medicine, Ramadi, Iraq

***Dear Editor,***

***This manuscript introduces a unique concept in an area that is both intriguing and important for medical research. The topic is timely and is likely to engage the journal's readers. Additionally, the manuscript is well-written, presenting ideas clearly and logically.***

***Dear Authors,***

***You are doing excellent work, and this manuscript reflects a great deal of effort and thoughtful research. The study is promising and contributes meaningfully to the field. However, there are a few important points that require further clarification and revision to enhance the overall quality and impact of the manuscript.***

***The introduction provides a comprehensive overview of PCOS and the hypothesised role of the KISS1 gene; however, the specific knowledge gap addressed by the current study should be stated more explicitly. While conflicting evidence regarding serum kisspeptin levels and KISS1 polymorphisms is mentioned, the introduction would benefit from a clearer explanation of why existing studies are insufficient and how the present study advances the field.***

***It is preferred to use the following reference to support your updated citations:*** ***(refer to 1)***

***The reported prevalence of PCOS (6%–10%) is appropriate; however, the diagnostic criteria used in cited studies (e.g., NIH, Rotterdam, or AES) are not specified. Given that PCOS prevalence varies considerably depending on diagnostic criteria, the authors should clarify this to improve interpretability and contextual accuracy.***

***Although the introduction discusses the physiological role of kisspeptin in GnRH and gonadotropin regulation, the mechanistic pathway linking KISS1 SNPs to PCOS pathogenesis remains somewhat speculative. A more detailed explanation of how specific polymorphisms may alter kisspeptin expression or function would strengthen the biological plausibility of the study hypothesis.***

***It is preferred to use the following reference to support your updated citations: (refer to 2)***

***The discussion of KNDy neurons is relevant but underdeveloped. The authors should expand on how alterations in neurokinin B, dynorphin, and kisspeptin signaling specifically contribute to PCOS-related neuroendocrine dysfunction and how this supports investigating KISS1 gene variants.***

***The final sentence states that the study determined the impact of KISS1 gene polymorphisms and haplotypes on PCOS development. This objective would benefit from greater specificity, including which polymorphisms or haplotypes were analyzed and whether the focus is on association, causation, or functional prediction.***

***It is preferred to use the following reference to support your updated citations:*** ***•   (refer to 3)***

***The manuscript states that 80 women with PCOS and 76 controls were initially recruited, but only 60 participants per group met the inclusion criteria. The specific reasons for exclusion (e.g., incomplete data, failure to meet diagnostic criteria, withdrawal of consent) should be explicitly described to enhance transparency and reproducibility.***

***Oligo- or anovulation was determined solely based on self-reported menstrual cycle frequency, which may be subject to recall bias. The authors should clarify whether menstrual history was corroborated with clinical records or supported by biochemical markers (e.g., mid-luteal progesterone), particularly given the diagnostic importance of ovulatory dysfunction in PCOS.***

***Although hyperandrogenism was defined using Ferriman–Gallwey scores and serum total testosterone levels, the manuscript lacks detail regarding:***

***The assessor training or blinding for Ferriman–Gallwey scoring, and***

***The assay method, timing, and reference ranges for testosterone measurement.***

***These details are essential for ensuring diagnostic consistency and comparability with other studies.***

***Participants were described as “self-reported as having Indonesian ethnic origin.” While this helps reduce population stratification bias, the authors should acknowledge the limitations of self-reported ethnicity, particularly in genetic association studies, and consider discussing whether sub-ethnic heterogeneity was assessed or controlled.***

***The Results section refers to comparisons between “lean and obese groups” and mentions “four groups”; however, this stratification is not clearly introduced or defined in the Methods or at the beginning of the Results. The authors should explicitly describe how subjects were categorized (e.g., BMI cutoffs) and ensure consistency between Methods, Tables, and Results.***

***The manuscript reports median ages and median kisspeptin levels but does not consistently indicate whether other variables are presented as means ± SE or medians (IQR). This inconsistency makes interpretation difficult. The authors should clarify which variables were normally distributed, justify the choice of statistical descriptors, and ensure alignment with the statistical tests used.***

***Although no statistically significant difference in kisspeptin levels was observed, the Results section still emphasizes the lower median value in the PCOS group. This observation should be reported cautiously and without implying biological relevance unless supported by statistical significance or effect size analysis.***

***There appears to be confusion in allele nomenclature, particularly for rs5780218 (e.g., reference to a “CT allele”). Alleles should be clearly distinguished from genotypes. The authors must revise this section to ensure correct and unambiguous genetic terminology.***

***It is preferred to use the following reference to support your updated citations:      (refer to 4 )***

***The Discussion appropriately acknowledges conflicting reports regarding serum kisspeptin levels in PCOS; however, the interpretation remains largely speculative. The authors should more clearly distinguish between central (hypothalamic) kisspeptin signalling and circulating kisspeptin levels, as serum measurements may not accurately reflect hypothalamic activity. This distinction is critical for reconciling discrepancies with studies reporting elevated kisspeptin expression in PCOS.***

***It is preferred to use the following reference to support your updated citations:*** ***(refer to 5)***

***Conclusion should be objective, with further perspective,e or should add at least a few sentences about future study/future perspective of .it***

Is the work clearly and accurately presented and does it cite the current literature?

Yes

If applicable, is the statistical analysis and its interpretation appropriate?

Yes

Are all the source data underlying the results available to ensure full reproducibility?

Yes

Is the study design appropriate and is the work technically sound?

Partly

Are the conclusions drawn adequately supported by the results?

Yes

Are sufficient details of methods and analysis provided to allow replication by others?

Yes

Reviewer Expertise:

Molecular Biology, Microbiology

I confirm that I have read this submission and believe that I have an appropriate level of expertise to confirm that it is of an acceptable scientific standard.

## References

[1] : Association of FokI, Tru91, and ApaI vitamin D receptor gene polymorphisms with the development of polycystic ovarian syndrome: A molecular genetic study. *Journal of King Saud University - Science* .2024;36(11) : 10.1016/j.jksus.2024.103563 10.1016/j.jksus.2024.103563

[2] : Cytogenetic screening of chromosomal abnormalities and genetic analysis of FSH receptor Ala307Thr and Ser680Asn genes in amenorrheic patients. *PeerJ* .2023;11: 10.7717/peerj.15267 10.7717/peerj.15267 PMC1022647737255590

[3] : Association Between NsiI and PmLI Insulin Receptors on the Development of Polycystic Ovarian Syndrome. *Pakistan Journal of Biological Sciences* .2024;27(11) : 10.3923/pjbs.2024.526.536 526-536 10.3923/pjbs.2024.526.536 39551955

[4] : The Association of Anti-Mullerian Hormone and Infertility Hormonal Imbalance with Polycystic Ovarian Syndrome Among Iraqi Patients. *Pakistan Journal of Biological Sciences* .2023;26(5) : 10.3923/pjbs.2023.241.248 241-248 10.3923/pjbs.2023.241.248 37859555

[5] : In terms of the PCR-RFLP technique, genetic screening of Ala575Val inactivating mutation in patients with amenorrhea. *Journal of Emergency Medicine, Trauma and Acute Care* .2022;2022(6) : 10.5339/jemtac.2022.aimco.8 10.5339/jemtac.2022.aimco.8

# Reviewer report: Reviewer response for version 1

**DOI:** 10.5256/f1000research.186221.r413858

**Published:** 2025-09-16

**Authors:** Mukhri Hamdan

**Affiliations:** 1 Universiti Malaya, Kuala Lumpur, Federal Territory of Kuala Lumpur, Malaysia

Congratulations on a great research report. It focuses on an upstream neuroendocrine gene (KISS1) with mechanistic plausibility, also including haplotype analysis,  Day 3–5 sampling attempt, and exclusion of recent HPG-altering medications with open data availability. 

 Here are the issues identified:

**Primary outcome specification. **The manuscript does not pre-specify a single primary genetic model/outcome; multiple inheritance models are tested across two SNPs (± haplotypes).

**Sample size and power**: No a priori power calculation is provided; small genotype cell counts (e.g., rs4889 GG) inflate ORs and widen CIs. Need to add an a priori (or post-hoc, with caveats) power analysis indicating detectable OR at α (after correction) for your MAF. Interpret large ORs from rare genotypes cautiously.

**Recruitment flow & attrition** recruited 80 PCOS and 76 controls, but analyzed 60+60; reasons for exclusion are not detailed. Can you provide a CONSORT-style flow diagram with reasons for exclusion and any differences between included vs excluded? 

**Group definition and stratification** Results text refers to “four groups” (lean vs obese strata), but Methods define only cases/controls; BMI cut-offs not specified. Please clearly define BMI strata (cut-points, rationale), when this stratification was planned, and where those subgroup results appear (tables/appendix). Align wording (two vs four groups).

**Normality, summary measures, and test. **Text says data are “mean ± SE” and “independent t-test” used, yet tables present medians (ranges), and several variables are non-normal. Please reassess distributions; report appropriate central tendency (mean±SD for normal; median[IQR] for skewed) and use Mann-Whitney U where needed. Ensure test choice matches what’s reported in tables.

**Genotyping quality control:** Limited QC details for Sanger calls (call rate, duplicate concordance, blinded repeats). Can you report per-SNP call rate, sample call rate, replicate concordance, and any re-reads? Include HWE p-values for **controls** (and for cases with caveat) and confirm all expected cell counts ≥5; use Fisher’s exact where violated.

**Population structure** “Indonesian ethnic origin” is self-reported in a genetically diverse setting. Please describe recruitment geography and, if available, adjust for population substructure (e.g., principal components). At a minimum, discuss the risk of stratification bias in limitations.

**Confounding adjustment** Logistic regression via SNPStats appears unadjusted for age, BMI, and insulin resistance. Please present adjusted models (age, BMI, HOMA-IR at a minimum). State covariate selection strategy and show both crude and adjusted ORs with 95% CIs.

**Haplotype and LD reporting.** Only D′ is reported; r² is not provided; haplotype inference power is limited with n=120.. Please add r² values, haplotype frequencies with CIs, and sensitivity analyses (e.g., EM convergence diagnostics). Discuss low-frequency haplotype instability. 

**Kisspeptin assay standardization. **Samples taken “day 3–5” may not be physiologically comparable in anovulatory PCOS; assay performance details (LoD, intra/inter-assay CVs, freeze-thaw cycles) are missing. Please provide preanalytical handling, kit performance metrics, and justify timing equivalence for PCOS vs controls; consider sensitivity analysis excluding uncertain cycle-phase samples.

**Diagnostic criteria implementation: **Hyperandrogenism definition mixes clinical (FG >8) and biochemical (T>60 ng/dL) using immunoassay (possible bias at low female T). Please clarify assay method limitations (vs LC-MS/MS), laboratory reference ranges, and how hirsutism scoring handles hair removal. Provide inter-rater reliability if available.

**Causal language** “Predisposing factor” overstates causality for a cross-sectional association. Please replace with “associated with increased odds of PCOS” throughout, including the title and conclusions. 

 That's all. Thank you

Is the work clearly and accurately presented and does it cite the current literature?

Yes

If applicable, is the statistical analysis and its interpretation appropriate?

I cannot comment. A qualified statistician is required.

Are all the source data underlying the results available to ensure full reproducibility?

Partly

Is the study design appropriate and is the work technically sound?

Yes

Are the conclusions drawn adequately supported by the results?

Partly

Are sufficient details of methods and analysis provided to allow replication by others?

Yes

Reviewer Expertise:

need to be a researcher working on the same field as this is too niche

I confirm that I have read this submission and believe that I have an appropriate level of expertise to confirm that it is of an acceptable scientific standard, however I have significant reservations, as outlined above.

# Response

**Published:** 2026-01-07

**Authors:** Gita Pratama

**Affiliations:** Obstetrics and Gynecology, Universitas Indonesia, Depok, West Java, Indonesia

Dear Reviewer,

 We sincerely thank you for your thorough, constructive, and insightful review of our manuscript entitled *“rs4889 and rs5782018 polymorphisms of KISS1 gene as genetic predisposing factor for PCOS in Indonesian women.”* We greatly appreciate your recognition of the study’s strengths and your detailed recommendations, which have substantially improved the rigor, clarity, and transparency of our work.

 Below, we provide a point-by-point response to each comment. 

 1. Primary outcome specification

 We agree with the reviewer. In the revised manuscript, we now explicitly state in the *Statistical Analysis* section that the **primary outcome** was the association between KISS1 polymorphisms (rs4889 and rs5780218) and PCOS status, evaluated under predefined inheritance models (codominant, dominant, recessive, and overdominant). Model selection was guided by **AIC and BIC**, which was prespecified as an exploratory approach rather than confirmatory hypothesis testing. We have clarified that results across multiple models should be interpreted as **exploratory**, and we have added a statement in the *Limitations* section regarding multiple testing and the absence of formal correction.

 2. Sample size and power considerations

 We acknowledge this limitation. As this was an exploratory candidate-gene study, no formal a priori power calculation was performed. In the revised manuscript, we have added a **post-hoc power estimation** (with explicit caveats) based on the observed minor allele frequencies and detectable odds ratios at α = 0.05.

 We further revised the *Results* and *Discussion* sections to emphasize **cautious interpretation of large ORs**, particularly for rare genotypes (e.g., rs4889 GG), and explicitly note the potential for overestimation due to small cell counts.

 3. Recruitment flow and attrition

 We thank the reviewer for this important observation. Reasons for exclusion included incomplete clinical data, withdrawal of consent, and failure to meet inclusion criteria upon reassessment. We have also clarified this information in the *Subjects* subsection of the *Methods* and confirmed that no significant demographic differences were observed between included and excluded participants.

 4. Group definition and stratification (lean vs obese)

 We agree and have corrected this inconsistency. BMI stratification (lean vs obese) is now **explicitly defined** in the *Methods* section using standard WHO cut-offs (BMI <25 kg/m² and ≥25 kg/m²). We clarify that BMI stratification was **preplanned for descriptive endocrine comparisons only** and not for primary genetic association testing. All references to “four groups” have been aligned with this clarification, and subgroup results are now clearly labeled in tables and text.

 5. Normality, summary measures, and statistical tests

 We have reassessed data distributions using normality testing. Variables with non-normal distributions are now presented as **median (IQR)**, and corresponding analyses were performed using **Mann–Whitney U tests**. Normally distributed variables are reported as **mean ± SD** with appropriate parametric testing.

 All tables and text have been revised to ensure consistency between reported summary measures and statistical tests.

 6. Genotyping quality control

 We have expanded the *Genotyping Methods* section to include detailed quality-control procedures, including:

- Per-SNP and per-sample call rates
- Duplicate sample concordance
- Manual chromatogram re-evaluation for ambiguous calls
- Hardy–Weinberg equilibrium testing in controls

We also clarify that **Fisher’s exact test** was applied where expected cell counts were <5.

 7. Population structure and stratification bias

 We acknowledge this limitation. In the revised manuscript, we describe recruitment geography (Jakarta and surrounding regions) and explicitly discuss the **risk of population stratification** due to Indonesia’s genetic diversity. As genome-wide markers were unavailable, adjustment using principal components was not feasible; this is now clearly stated as a limitation.

 8. Confounding adjustment

 We have now included **multivariable logistic regression analyses** adjusting for age, BMI, and HOMA-IR. Both crude and adjusted odds ratios with 95% confidence intervals are presented in the revised results. Covariate selection was based on established PCOS risk factors and biological plausibility, as now stated in the *Statistical Analysis* section.

 9. Haplotype and linkage disequilibrium reporting

 We have added **r² values** alongside D′ to provide a more complete LD assessment. Haplotype frequencies with confidence intervals are now reported, and we discuss the **instability of low-frequency haplotypes** and limited inference power given the sample size.

 10. Kisspeptin assay standardization and cycle timing

 We have expanded the *Hormone Measurements* section to include assay performance characteristics (limit of detection, intra- and inter-assay CVs, sample handling, and freeze–thaw procedures). 

 11. Diagnostic criteria and androgen measurement

 We have clarified the definition of hyperandrogenism, acknowledged the limitations of immunoassay-based testosterone measurement compared with LC-MS/MS, and provided laboratory reference ranges. The handling of cosmetic hair removal in Ferriman–Gallwey scoring is now described, and inter-rater consistency procedures are noted where applicable.

 12. Causal language in title and conclusions

 We fully agree and have revised the **results interpretation and conclusions** to replace causal language with **“associated with increased odds of PCOS.”** This change has been applied consistently throughout the manuscript.

 Once again, we thank the reviewer for their thoughtful and detailed critique. We believe these revisions substantially strengthen the methodological transparency and interpretability of our study, and we hope the revised manuscript satisfactorily addresses all concerns raised.

 Sincerely,

 Authors
